# The path to sustainable cardiac surgery in Rwanda: analysis of costs for consumables used during cardiac surgery for a non-governmental organization

**DOI:** 10.1186/s13019-024-03087-x

**Published:** 2024-10-01

**Authors:** Hannah Rando, Maurice Musoni, Bonnie C. Greenwood, Lambert Ingabire, Sam Van Hook, Ceeya Patton Bolman, R. Morton Bolman, Yihan Lin

**Affiliations:** 1 Division of Cardiac Surgery, Department of Surgery, Johns Hopkins University School of Medicine, Baltimore, MD USA; 2 Team Heart, Inc, Milton, MA USA; 3 Division of Cardiac Surgery, Department of Surgery, King Faisal Hospital, Kigali, Gasabo Rwanda; 4 Department of Pharmacy, Lahey Hospital and Medical Center, Burlington, MA USA; 5 Department of Pharmacy, King Faisal Hospital, Kigali, Gasabo Rwanda; 6 Division of Cardiothoracic Surgery, Department of Surgery, University of Colorado Anschutz Medical Campus, Aurora, CO USA; 7 Division of Cardiothoracic Surgery, Department of Surgery, University of Minnesota, Minneapolis, MN USA; 8 Division of Cardiothoracic Surgery, Department of Surgery, Stanford University School of Medicine, Palo Alto, CA USA; 9 Department of Surgery, University of Vermont Medical Center, 111 Colchester Ave, Burlington, VT 05408 USA

**Keywords:** Costs and cost analysis, Cardiac surgical procedures, Developing countries, Global health

## Abstract

**Background:**

Until local healthcare infrastructure is strengthened, cardiac surgical care in low- and middle-income countries is often provided by non-governmental organizations by way of visiting healthcare teams. This is generally considered to be a cost-effective alternative to transporting patients to high income countries for surgical care, but the costs of cardiac surgery consumables under this model are poorly understood. Our objective was to identify the per-patient cost of cardiac surgery consumables used in single and double valve replacements performed by a non-governmental organization in Rwanda.

**Methods:**

Financial data from 2020 were collected from Team Heart, a non-governmental organization that supports cardiac surgical care in Rwanda. A comprehensive list of consumables was generated, including surgical, perfusion, anesthesia, and inpatient supplies and medications. Acknowledging the variability in perioperative needs, the quantities of consumables were calculated from an average of six patients who underwent single or double-valve replacement in 2020. Total costs were calculated by multiplying purchasing price by average quantity per patient. Costs absorbed by the local hospital were excluded from the calculations.

**Results:**

The total cost per patient was estimated at $9,450. Surgical supplies comprised the majority of costs ($6,140 per patient), with the most substantial cost being that of replacement valves ($3,500 per valve), followed by surgical supplies ($1,590 per patient).

**Conclusions:**

This preliminary analysis identifies a cost of just over $9,000 per patient for consumables used in cardiac valve surgery in Rwanda, which is lower than the estimated costs of transporting patients to centers in high income countries. This work highlights the relative cost effectiveness of cardiac surgical care in low- and middle- income countries under this model and will be instrumental in guiding the allocation of local and international resources in the future.

**Supplementary Information:**

The online version contains supplementary material available at 10.1186/s13019-024-03087-x.

## Background

Access to surgical care in low- and middle-income countries (LMICs) is wholly inadequate, with nine out of ten individuals, or a total of over 5 billion people, lacking access to basic surgical services [1]. While this issue pertains to all surgical subspecialties, it is perhaps even more notable in the area of cardiac surgery. More than 100 countries lack a single cardiac surgeon, and low-income countries average only 0.04 cardiac surgeons per million individuals, compared to 7.15 cardiac surgeons per million individuals in high-income countries [2]. These disparities are even more notable when considering the overall disease burden within LMICs; cardiovascular diseases are the leading cause of morbidity and mortality worldwide, and an estimated one-third of patients will require surgical or interventional care [2]. The development of self-sustainable in-country cardiac surgery programs offers the best long term solution to these disparities, but this requires substantial financial and personnel investments that may not be immediately available [3, 4]. In the interim, solutions to address cardiac surgical disease in LMICs have included transportation of patients to high income countries (HICs) with established cardiac surgery centers, and short term humanitarian missions wherein cardiac surgical procedures are performed in-country by visiting teams of trained cardiac surgical staff.

Rwanda represents one such LMIC with disparate access. Prior to 2020, when Rwanda’s first cardiac surgeon completed his training, cardiac surgical care in Rwanda was available exclusively through expatriate teams and was therefore limited to only a small number of patients each year [2]. In acknowledgement of this deficit, a non-governmental organization (NGO) called Team Heart was founded in 2006. The organization was established in conjunction with the Rwandan Ministry of Health and offered humanitarian cardiac surgery performed by visiting teams with the simultaneous objective of mentoring local medical professionals to build a self-sustainable cardiac surgery program [5]. 

When considering this type of hybrid model for cardiac surgical care, wherein a gradual transition is made to local independence, an integral component is understanding the costs associated with cardiac surgery performed by visiting teams. Surgical supplies used by nonprofit organizations are frequently donated by external organizations or offered at a discounted rate, which can make defining the true costs more challenging. Furthermore, changes in supply availability and shifts in local and international supply chains can cause frequent fluctuations in the costs associated with cardiac surgical care [6]. Our objective was to estimate costs of cardiac surgery consumables used by Team Heart to inform future humanitarian efforts and promote additional economic support to this critical area of global health.

## Methods

We performed a retrospective review of the financial records from Team Heart, an NGO that facilitated the majority of adult cardiac valvular operations in Rwanda between 2006 and 2022. From these records, we generated a comprehensive list of supplies and medications utilized regularly in cardiac valve operations for rheumatic heart disease. All operations were performed at King Faisal Hospital in Kigali. King Faisal is a 160-bed hospital with over 8,000 admissions each year, and is the largest referral hospital in Rwanda. It is currently the only hospital in Rwanda where cardiac surgery is offered. The study design and methods for data collection were approved by the Rwanda National Ethics Committee and need for informed consent was waived due to the retrospective nature of the study.

After generating a list of consumables, the purchasing price for each supply and medication was identified. Purchasing price was defined as the most recent price paid by the NGO (in US dollars). All supplies were purchased from manufacturers in the United States and Europe, with the exception of cardiac valves, which were donated to the organization by a United States based manufacturer. Purchasing price for replacement valves was instead estimated using an expert consensus from a group of four surgeons. Data were categorized by phase of care (anesthesia, perfusion, surgical, and postoperative inpatient). Medications and fluids used across all phases of care were reported separately.

After the list of supplies and their corresponding costs was finalized, we prospectively collected the quantity of each supply and medication used for patients who received a cardiac valve operation in a single service trip in 2020. Average quantity of each item used per case was then calculated, and total costs were then estimated by multiplying purchasing price by average quantity used per case. Individual patient characteristics and hospital outcomes were also collected to contextualize the costs reported herein. Patient data were reported using summary statistics, including mean and standard deviation or median and interquartile range for continuous data, and percentages for categorical data.

## Results

### Surgical cases

A total of six cardiac surgery cases were observed to estimate the quantity of each medication and supply used per patient. All cases were performed for rheumatic heart disease and included a minimum of one valve replacement. All valves placed were mechanical valves. There were a total of three aortic valve replacements, five mitral valve replacements, and one tricuspid valve repair (Table 1). Patients were generally young (median age 29 years, IQR 23–37) with preserved left ventricular function before and after surgery (median preoperative LVEF 55%, IQR 45–60%; median postoperative LVEF 45%, IQR 41–53%). Half of the patients developed postoperative atrial fibrillation. There were otherwise no postoperative complications or deaths. Median duration of hospitalization was 12 days (IQR 11.5–14 days).

**Table 1:** Patient characteristics

Age	29 (23–37)
Preoperative LVEF (%)	55 (45–60)
≥50%	3 (50)
40–49%	2 (33)
30–39%	0 (0)
<30%	1 (17)
Preoperative pulmonary hypertension	
None	3 (50)
Mild	1 (17)
Moderate	1 (17)
Severe	1 (17)
Procedure	
AVR	1 (17)
MVR	2 (33)
AVR/MVR	2 (33)
MVR/TVR	1 (17)
Cross clamp time, minutes	98 (91–133)
Cardiopulmonary bypass time, minutes	126 (117–175)
Postoperative LVEF (%)	45 (41–53)
≥50%	2 (33)
40–49%	2 (33)
30–39%	1 (17)
<30%	1 (17)
Duration of hospitalization, days	12 (11.5–14)
Postoperative complications	
No complication	3 (50)
Atrial fibrillation	3 (50)
Prolonged ventilatory support	0 (0)
Renal failure	0 (0)
Reoperation	0 (0)
Stroke	0 (0)
Mortality	0 (0)

Perioperative characteristics of patients who underwent single- or double-valve replacement in Rwanda. Data are reported as mean (IQR) or n(%). Prolonged ventilatory support was defined as > 24 h. LVEF = left ventricular ejection fraction. AVR = aortic valve replacement. MVR = mitral valve replacement. TVR = tricuspid valve repair

### Cost categories

Anesthesia supplies: The average cost of anesthesia supplies was $444 per patient (Supplementary Table 1). The highest anesthesia supply cost was for central line kits.

Perfusion supplies: Perfusion supplies cost an average of $1,570 per patient (Supplementary Table 2).

Surgical supplies: Supplies used in the operating room, not including those incurred by anesthesia, averaged $1,590 per patient (Supplementary Table 3). The most substantial surgical costs were the costs of the cardiac surgical pack, hemostatic agents (e.g. Surgicel), chest tubes, and valve sutures (e.g. Ethibond).

Replacement valves: The estimated cost per surgical valve was $3,500. For our sample of six patients, the average number of valves replaced was 1.33, resulting in an average cost of $4,550 per patient.

Postoperative inpatient supplies: Costs of inpatient supplies, not including medications, averaged $272 per patient (Supplementary Table 4).

Medications and fluids: Average costs of medications and fluids, including consumables used both intraoperatively and postoperatively, was $1,030 per patient. The most expensive medication was protamine, estimated at $92 per patient (Supplementary Table 5).

Total costs: Total average cost per patient, including anesthesia, perfusion, and surgical supplies, inpatient costs, medications, IV fluids, and cardiac valves was $9,450 (Table 2).

**Table 2:** Total estimated costs in US dollars

Category	Total Cost (USD)
Anesthesia supplies	444.26
Perfusion supplies	1,570.09
Surgical supplies	1,587.35
Inpatient supplies	271.62
Medications	468.01
Fluids	564.47
Replacement valves	4,550.00
**Total**	**9**,**455.80**

Estimated costs of consumables for single- and double-valve replacements in Rwanda. Replacement valves were estimated at a cost of $3,500 per valve, with an average of 1.3 valves per patient. USD = United States dollars

## Discussion

As local cardiac surgery programs are developed, NGO-funded medical missions are often an intermediate step in the delivery of cardiac surgical care, but the costs of cardiac surgical care under this model are poorly understood. To address this deficit, our objective was to estimate the cost of cardiac surgery consumables used by an NGO for single and double valve replacements in Kigali, Rwanda. We found that the average cost incurred was just over $9,000 per patient, with over one third of the cost attributed to the replacement valve itself.

### Comparison of costs with HICs and other LMICs

It is important to interpret these cost estimates within the context of other available options for cardiac surgical care in LMICs. In the absence of a full time local program, the primary alternative is to transport patients with cardiac surgical disease to HICs with established programs. The cost to do so is somewhat variable depending on the destination program and cost of travel, but NGOs that offer this option estimate a cost of $15,000-$25,000 per patient [7, 8]. In contrast, a cost of $9,000 per patient is not only more affordable, but also confers the simultaneous benefit of training and mentoring local teams.

In Rwanda in particular, healthcare costs are covered primarily by Mutuelles de Santé, a universal health insurance program where member premiums and copays are adjusted based on socioeconomic status and costs are shared by the Rwandan government, international organizations, foreign charities, and member premiums [9]. In total, 62% of healthcare funding comes from abroad and 38% comes from domestic funds [10]. As such, investing in the most cost effective strategy for cardiac surgical care not only benefits the patients and local government, but also benefits international stakeholders.

Comparing these costs to those anticipated with a fully established in-country program with locally sourced supplies is more of a challenge, given the substantial variability of cardiac surgery costs seen across the globe. In a recent review published by Vervoort et al., estimated costs in LMICs ranged from $2,000 in India, up to $11,000 in Nigeria [11, 12], with each study varying considerably in the methodology used. This degree of inconsistency in both reported cost and methodology emphasizes the inability to extrapolate costs between LMICs and the difficulty in defining costs in a setting often marked by unreliable supply chains and fluctuations in pricing [6]. 

Perhaps the most relevant comparison for Rwanda is the publication written by Falase et al., in which costs were calculated for a single center in Nigeria, which at the time was the only center in the country that offered cardiac surgical services [13]. The authors similarly catalogued costs as either theatre, perfusion, medication, intensive care, hospital stay, or investigation costs. Although costs were outlined for several common cardiac procedures, the average cost of mitral valve replacement was the most expensive, at $11,200. On initial evaluation, one might conclude that cardiac valvular surgery in Rwanda is less costly, but Falase et al. included pre- and post-operative investigations (e.g. echocardiography) and the cost of the hospitalization- both of which our study was unable to account for. Conversely, the authors accounted for only single valve replacement, whereas our estimates were partially calculated from double valve replacements. When adjusting for these differences, the estimated cost of mitral valve replacement in Nigeria is roughly $1,000 less than our estimates in Rwanda. Given that Falase et al. calculated costs using locally sourced supplies, this may reflect a promising trend for Rwanda in the future, as supplies are increasingly procured from domestic sources. Although the data to support this hypothesis is currently lacking within cardiac surgery specifically, numerous other global public health interventions have seen a trend towards lower costs when using locally obtained resources, suggesting that the cost effectiveness of cardiac surgery interventions will continue to improve as local infrastructure is strengthened [14–16]. 

### Local context

Since the first Rwandan cardiothoracic surgeon completed his training and returned to King Faisal Hospital to practice, the local staff has grown and has gained progressive clinical independence and confidence. Historically, fewer than 30 cardiac operations were performed each year in Rwanda, as procedures occurred exclusively during trips by visiting NGOs. As recently as 2019, there was only one cardiothoracic surgeon in Rwanda, and all cases were performed with support from visiting teams [17]. There has been exponential growth of the cardiac surgery program since that time; the cardiac surgery team now operates independently and consists of four cardiac surgeons, two cardiac anesthesiologists, four adult cardiologists, two pediatric cardiologists, four perfusionists, four operating room nurses, and ten cardiac intensive care unit (ICU) nurses. Recruitment and training have been heavily prioritized, and many team members have completed or are undergoing training abroad in Kenya, India, Israel, France, and Vietnam. A cardiology fellowship program has been developed and will be graduating its first cohort this year. Fellowship programs in cardiac surgery, cardiac anesthesia, critical care, and pulmonology are all under development.

Even so, a substantial proportion of material support comes from international donors, there are ongoing equipment needs, and there are a limited number of operating rooms and ICU beds which has limited the number of cases performed each year. The positive developments in staffing call for increased funding to support programmatic growth and address the currently unmet population needs. As the program continues to expand, we would suggest prioritization of funding to the development of local supply chains, investment in capital equipment, expansion of operating and ICU space, and funding scholarships for the training and recruitment of a skilled workforce.

### Global context

This progressive upscaling of efforts in Rwanda comes at a critical time for global cardiac surgery. In 2017, societal leaders from around the world gathered in Cape Town South Africa and developed the “Cape Town Declaration on Access to Cardiac Surgery in the Developing World” [18]. The declaration emphasized the importance of addressing rheumatic heart disease in the developing world and led to establishment of the Cardiac Surgery Intersociety Alliance (CSIA)- a collaboration between The Society of Thoracic Surgeons, American Association for Thoracic Surgery, the European Association for Cardio-Thoracic Surgery, the Asian Society for Cardiovascular and Thoracic Surgery, and the World Heart Federation. Since its genesis, the CSIA has begun to identify and assist young cardiothoracic surgery programs, with support administered to programs in Rwanda and Mozambique, to date [19]. This large scale NGO support offers a promising and cost-effective method for supporting developing programs by providing scholarships for training of cardiac surgical team members and encouraging “South-South Cooperation” in cardiac surgery with exchange of resources, knowledge, and technology.

As additional programs are initiated and supported in other LMICs, the data described herein will be integral to inform the degree of financial support needed and the feasibility of performing cardiac surgery with external support. The estimated costs of consumables are applicable not only to other NGOs considering similar work, but also to young programs without established supply chains that remain reliant on outsourced products. Furthermore, our data highlights the substantial cost of cardiac valves themselves relative to the total cost of the operation. As new cardiac surgery programs are established around the globe, an important step in sustainability will be to support the simultaneous development of local and affordable cardiac valve manufacturing.

### Limitations and future directions

Our analysis had several limitations. Most importantly, our analysis represents only the costs of consumables incurred by an NGO, and should not be viewed as a comprehensive analysis of all cardiac surgical costs. Operative trips by Team Heart require substantial volunteer efforts, with each trip including a minimum of two cardiac surgeons, two anesthesiologists, two perfusionists, one pharmacist, seven nurses, one cardiologist, and one sonographer (Table 3). The costs of averted labor and travel expenses for these personnel were not included, as these costs were absorbed by the volunteers, not the organization. The average flight from the United States to Rwanda costs roughly $1,750, which substantially increases the total per-patient costs of conducting cardiac surgery with NGO support. Furthermore, investing in a new cardiac surgery program initially requires a surplus of staffing, as both volunteer and local healthcare workers are involved in patient care. The salaries of the local healthcare workers trained during operative trips by the NGO were not accounted for, nor were the indirect costs absorbed by the local hospital (e.g. administration, operations, building repairs and maintenance, equipment, fuel, electricity, water, and space). Future studies that comprehensively evaluate cardiac surgical costs using local resources and including costs to the local hospital will be of great value in the discussion of the relative cost effectiveness of cardiac surgical care in LMICs.

There are also limitations to the generalizability of our data. Costs of replacement valves were estimated from an expert consensus and may not be generalizable to all manufacturers. We were also only able to account for the costs of cardiac valve procedures, which may not be representative of all cardiac surgical procedures in Rwanda. Similarly, the small sample size and heterogeneity in procedures results in individual data points heavily influencing the summary measures, which may limit the validity and generalizability of the data. Finally, costs were calculated from a relatively stable cohort of patients, and may not reflect the costs of more complex procedures. Investigations that include a larger number of patients and a wider variety of cardiac surgical procedures may yield a more reliable cost estimate.

Regardless, this preliminary analysis serves as the framework for future studies and provides a reasonable estimate of cardiac surgical costs to an NGO, which will be invaluable to the country and to other young cardiothoracic surgery programs across the globe. We anticipate that subsequent research performed in partnership with local stakeholders will demonstrate comparable or reduced costs using local resources and will validate the feasibility of cardiac surgery in a low resource setting.

**Table 3:** Unaccounted costs

Cost	Components
Personnel	
Anesthesiologist	2 anesthesiologists
Surgeon	2 surgeons
Perfusionist	2 perfusionists
Scrub nurse	1 scrub nurse
Cardiologist	1 cardiologist
Intensivist	2 intensivists
Nurse (ICU/floor)	7 nurses
Pharmacist	1 pharmacist
Preoperative workup	
Radiology evaluation	Chest x-ray, echocardiogram
Laboratory evaluation	Type and screen, HIV, hepatitis, BMP, LFTs, CBC, coagulation studies
Transportation	Transportation to and from appointments
Postoperative follow up	
Radiology evaluation	Echocardiogram
Consultations	Cardiology, cardiac surgery
Medications	Furosemide, warfarin
Transportation	Transportation to and from appointments
Indirect costs	
Electricity/Fuel/Water	Total used in OR, ICU, and floor spaces dedicated to cardiac services
Equipment	All major and minor equipment used for service delivery
Maintenance	Supplies and personnel needed for repairs and renovations
Administration	Operational and administrative costs dedicated to cardiac services

List of additional costs involved in conducting cardiac surgery that were not accounted for by our current analysis. BMP = basic metabolic panel; LFTs = liver function tests; CBC = complete blood count

## Electronic supplementary material

Below is the link to the electronic supplementary material.

Supplementary Material 1

## Data Availability

No datasets were generated or analysed during the current study.

## Declarations

### Meeting presentation

This research was presented in part at the 103rd Annual Meeting of the American Association for Thoracic Surgery in Los Angeles, California, May 6^th^-9^th^, 2023.

## Abbreviations

CSIA: Cardiac Surgery Intersociety Alliance
HIC: High income countries
LMIC: Low and middle-income countries
LVEF: Left ventricular ejection fraction
NGO: Non-governmental organization
