# Supplementary material for: The path to sustainable cardiac surgery in Rwanda: analysis of costs for consumables used during cardiac surgery for a non-governmental organization
Source: J Cardiothorac Surg. 2024 Oct 1;19:574. doi: 10.1186/s13019-024-03087-x (PMC11443655; doi:10.1186/s13019-024-03087-x)
Supplement: Supplementary file 1 — Supplementary Material 1 [file 13019_2024_3087_MOESM1_ESM.docx]

Supplementary Table 1: Anesthesia supplies and costs in US dollars

| Anesthesia Supply | Quantity | Item Cost (USD) | Total Cost (USD) |
| --- | --- | --- | --- |
| 3-way stopcocks | 4 | 3.91 | 15.64 |
| Alcohol wipes | 20 | 0.03 | 0.60 |
| Anesthesia circuit | 1 | 11.54 | 11.54 |
| Angiocath, 18 gauge | 1 | 8.47 | 8.47 |
| Angiocath, 20 gauge | 1 | 8.47 | 8.47 |
| Arterial line kit | 1 | 73.56 | 73.56 |
| Bite blocks | 1 | 3.04 | 3.04 |
| Blood transfusion set | 1 | 7.89 | 7.89 |
| Claves | 3 | 2.80 | 8.40 |
| Central line kit, triple-lumen | 1 | 188.47 | 188.47 |
| EKG electrodes | 8 | 0.30 | 2.40 |
| Endotracheal tube | 1 | 3.52 | 3.52 |
| Endotracheal tube suction catheter | 1 | 0.26 | 0.26 |
| IV start kits | 3 | 2.53 | 7.59 |
| IV tubing | 2 | 2.29 | 4.58 |
| Nasogastric tube, 16 French | 1 | 10.77 | 10.77 |
| Needles | 10 | 0.27 | 2.70 |
| Non-sterile gloves | 10 | 0.31 | 3.31 |
| Oral airway | 1 | 14.61 | 14.61 |
| Pressure bag | 1 | 13.11 | 13.11 |
| Sterile drape | 1 | 8.20 | 8.20 |
| Sterile gown | 3 | 4.62 | 13.86 |
| Sterile gloves | 1 | 1.14 | 1.14 |
| Syringe, 3 cc | 2 | 0.28 | 0.56 |
| Syringe, 5 cc | 2 | 0.34 | 0.68 |
| Syringe, 10 cc | 10 | 0.33 | 3.33 |
| Syringe, 20 cc | 4 | 0.61 | 2.44 |
| Syringe, 50 cc | 4 | 1.02 | 4.08 |
| Syringe caps | 15 | 0.31 | 4.65 |
| Ultrasound cover | 1 | 1.25 | 1.25 |
| Yankauer | 3 | 5.06 | 15.18 |

*Estimated quantities and costs of anesthesia supplies used for single- and double-valve replacements in Rwanda. Quantity was calculated as an average from six surgical cases. USD= United States dollars. IV= intravenous.*

Supplementary Table 2: Perfusion supplies and costs in US dollars

| Perfusion Supply | Quantity | Item cost (USD) | Total Cost (USD) |
| --- | --- | --- | --- |
| ABG syringe | 3 | 1.38 | 4.14 |
| ACT cartridge | 10 | 2.29 | 22.90 |
| Aortic cannula | 1 | 50.35 | 50.35 |
| Cell salvage | 1 | 178.20 | 178.20 |
| Coronary ostial cannula | 1 | 40.21 | 40.21 |
| Coronary perfusion adaptor | 1 | 2.59 | 2.59 |
| Gloves, non-sterile | 8 | 0.33 | 2.64 |
| Hemoconcentrator | 1 | 121.50 | 121.50 |
| Left heart vent | 1 | 25.18 | 25.18 |
| Level detector | 1 | 0.25 | 0.258 |
| Pericardial sump | 1 | 11.01 | 11.01 |
| Pressure monitoring extension | 1 | 8.84 | 8.84 |
| Retrograde cannula | 1 | 90.63 | 90.63 |
| Syringe, 3 cc | 10 | 0.28 | 2.80 |
| Syringe, 6 cc | 2 | 0.33 | 0.66 |
| Syringe, 35 cc | 2 | 1.04 | 2.08 |
| Tubing pack with oxygenator | 1 | 927.37 | 927.37 |
| Venous cannula, dual stage | .17 | 33.37 | 5.00 |
| Venous cannula, single stage | 1.67 | 77.28 | 129.06 |

*Estimated quantities and costs of perfusion supplies used for single- and double-valve replacements in Rwanda. Quantity was calculated as an average from six surgical cases. USD= United States dollars. ABG= arterial blood gas. ACT= activated clotting time.*

Supplementary Table 3: Surgical supplies and costs in US dollars

| Surgical Supply | Quantity | Item cost (USD) | Total Cost (USD) |
| --- | --- | --- | --- |
| Aluminum blanket | 1 | 2.15 | 2.15 |
| Bone wax | 1 | 27.97 | 27.97 |
| Bovie grounding pad | 1 | 9.58 | 9.58 |
| Cardiac surgical pack | 1 | 202.62 | 202.62 |
| Chest tube, 24 French | 2 | 60.12 | 120.24 |
| Defibrillator pads, set of 2 | 1 | 33.29 | 33.29 |
| Dermabond | 1 | 76.08 | 76.08 |
| Dura prep | 1 | 11.28 | 11.28 |
| Ethibond suture, 2-0 | 3 | 63.06 | 189.20 |
| Foley kit | 1 | 16.41 | 16.41 |
| Gabbay frater | 1 | 47.31 | 47.31 |
| Hibiclens chlorhexidine | 0.3 | 7.73 | 2.32 |
| Ioban steri-drape | 1 | 41.88 | 41.88 |
| Laparotomy pads | 2 | 0.27 | 0.54 |
| Ligating clips | 1 | 17.80 | 17.80 |
| Monocryl, 3-0 | 1 | 30.94 | 30.94 |
| Non-sterile gloves | 15 | 0.33 | 4.95 |
| Pleur-evac | 1 | 92.63 | 92.63 |
| Prolene suture, 4-0 | 4 | 5.08 | 20.32 |
| Prolene suture, 3-0 | 5 | 5.02 | 25.10 |
| Shoe covers | 10 | 0.91 | 9.10 |
| Silk suture, 0 | 2 | 2.00 | 4.00 |
| Silk ties, 0 | 2 | 1.24 | 2.48 |
| Silk tape | 0.2 | 2.95 | 0.59 |
| Sterile gloves | 9 | 3.52 | 31.66 |
| Sternal saw blades | 1 | 15.00 | 15.00 |
| Sternal wires, set of 4 | 1 | 83.05 | 83.05 |
| Surgical caps | 4 | 0.36 | 1.44 |
| Surgical gowns | 7 | 4.62 | 32.34 |
| Surgical masks | 10 | 0.94 | 9.40 |
| Surgicel | 1 | 197.00 | 197.00 |
| Tegaderm | 4 | 2.72 | 10.88 |
| Telfa | 1 | 0.04 | 0.04 |
| Umbilical tape | 2 | 7.56 | 15.12 |
| Vascular tourniquet | 2 | 3.00 | 6.00 |
| Ventricular pacing wires | 2 | 42.73 | 87.46 |
| Vicryl suture, 0 | 2 | 50.35 | 100.70 |
| Y-connector | 1 | 9.83 | 9.83 |

*Estimated quantities and costs of surgical supplies used for single- and double-valve replacements in Rwanda. Quantity was calculated as an average from six surgical cases. USD= United States dollars.*

Supplementary Table 4: Inpatient supplies and costs in US dollars

| Inpatient Supply | Quantity | Item cost (USD) | Total Cost (USD) |
| --- | --- | --- | --- |
| Claves | 5 | 2.80 | 14.00 |
| EKG electrodes | 2 | 0.30 | 0.60 |
| End caps | 10 | 0.22 | 2.20 |
| Endotracheal tube holders | 1 | 5.64 | 5.64 |
| Face masks | 1 | 2.42 | 2.42 |
| Gauze, 3x3 | 15 | 0.10 | 1.50 |
| Gloves, sterile | 2 | 0.38 | 0.76 |
| Gloves, non-sterile | 20 | 0.33 | 6.60 |
| Incentive spirometer | 1 | 8.18 | 8.18 |
| IV tubing | 2 | 2.29 | 4.58 |
| Labels | 8 | 0.12 | 0.96 |
| Medicine cups | 15 | 0.01 | 0.15 |
| Pleur-evac | 1 | 92.63 | 92.63 |
| Suction catheter | 2 | 0.52 | 1.04 |
| Suction tubing | 5 | 1.89 | 1.89 |
| Suture removal kit | 2 | 1.49 | 2.98 |
| Syringe, ABG | 5 | 0.90 | 4.50 |
| Syringe, 3 mL | 10 | 0.28 | 2.80 |
| Syringe, 10 mL | 5 | 0.33 | 1.65 |
| Syringe, 50 mL | 10 | 1.04 | 10.40 |
| Saline flush | 60 | 1.06 | 63.60 |
| Tegaderm, small | 5 | 0.84 | 4.20 |
| Thermometer | 1 | 4.16 | 4.16 |
| Toothbrush | 1 | 1.48 | 1.48 |
| Toothpaste | 1 | 0.44 | 0.44 |
| Vacutainers | 5 | 1.66 | 8.3 |
| Vaseline gauze | 5 | 0.86 | 4.30 |
| Y-connector | 2 | 9.83 | 19.66 |

*Estimated quantities and costs of perfusion supplies used for single- and double-valve replacements in Rwanda. Quantity was calculated as an average from six surgical cases. USD= United States dollars. EKG= electrocardiogram. ABG= arterial blood gas.*

Supplementary Table 5: Medications and costs in US dollars

| Medication | Quantity | Item Cost (USD) | Total Cost (USD) |
| --- | --- | --- | --- |

| Adenosine 3 mg/mL; 2 mL vial | 0.5 | 7.76 | 3.88 |
| --- | --- | --- | --- |
| Adrenaline 1 mg/mL; 1 mL amp | 6.4 | 0.25 | 1.61 |
| Amiodarone 200 mg tab | 5 | 0.24 | 1.22 |
| Amiodarone 50 mg/mL; 3mL vial | 1 | 3.88 | 3.88 |
| Artemether/lumefantrine 20/120 mg tab | 18 | 0.17 | 3.04 |
| Aspirin 75 mg tab | 10 | 0.06 | 0.62 |
| Atenolol 25-50 mg tab | 7 | 0.02 | 0.12 |
| Atropine 1 mg/mL; 1mL amp | 0.5 | 0.78 | 0.39 |
| Bisacodyl 5 mg tab | 30 | 0.42 | 12.57 |
| Calcium gluconate 1 mg/mL; 10 mL vial | 10 | 1.09 | 11.41 |
| Captopril 12.5-25 mg tab | 15 | 0.01 | 0.20 |
| Carvedilol 12.5 mg tab | 5 | 0.17 | 0.83 |
| Cefazolin 1000 mg vial | 4 | 3.64 | 14.57 |
| Desmopressin 4 mcg/mL; 1 mL amp | 0.5 | 29.43 | 14.71 |
| Dextrose 50%; 50 mL vial | 0.1 | 0.81 | 0.08 |
| Digoxin 250 mcg tab | 2.5 | 0.33 | 0.83 |
| Digoxin 250 mcg/mL; 2 mL amp | 0.25 | 4.66 | 1.16 |
| Diltiazem 30 mg tab | 2.5 | 1.09 | 2.72 |
| Dobutamine 250 mg/20 mL vial | 0.7 | 15.52 | 10.87 |
| Enalapril 5 mg tab | 7 | 0.05 | 0.34 |
| Ephedrine 50 mg/mL vial | 1.25 | 1.16 | 1.46 |
| Esmolol 10 mg/mL; 10 mL vial | 0.25 | 6.02 | 1.50 |
| Fentanyl 50 mcg/mL; 2 mL vial | 2 | 0.84 | 1.69 |
| Fentanyl 500 mcg/mL; 2 mL vial | 2 | 2.22 | 4.43 |
| Furosemide 10 mg/mL; 2 mL vial | 10 | 0.21 | 2.12 |
| Furosemide 20-40 mg tab | 30 | 0.02 | 0.58 |
| Glyceryl trinitrate spray | 0.05 | 16.53 | 0.83 |
| Heparin 5,000 unit/mL; 5 mL vial | 5 | 6.99 | 34.93 |
| Hydralazine 20 mg/mL vial | 0.1 | 2.17 | 0.22 |
| Hydralazine 25 mg tab | 2.5 | 0.19 | 0.49 |
| Hydrocortisone 100 mg/2mL vial | 0.2 | 0.47 | 0.09 |
| Insulin regular 100 units/mL; 3mL vial | 0.5 | 4.35 | 2.17 |
| Isoflurane 100 mL | 0.05 | 49.67 | 2.48 |
| Ketamine 50 mg/mL; 10 mL | 1 | 2.47 | 2.47 |
| Labetalol 5 mg/mL; 20 mL amp | 0.25 | 34.47 | 8.62 |
| Lactulose | 0.05 | 4.07 | 0.20 |
| Lidocaine 2%; 5 mL | 2 | 0.42 | 0.84 |
| Lidocaine 2%; 20 mL | 1.2 | 4.86 | 5.83 |
| Magnesium oral | 2 | 2.17 | 5.43 |
| Magnesium sulfate 10%; 10 mL | 4 | 1.24 | 4.97 |
| Magnesium sulfate 50%; 10 mL vial | 4 | 0.90 | 3.59 |
| Methylene blue 10%; 5 mL vial | 0.05 | 1.31 | 0.07 |
| Metoclopramide 10 mg/2 mL vial | 0.5 | 0.23 | 0.12 |
| Metoprolol 5 mg/5 mL vial | 0.5 | 7.53 | 3.76 |
| Metoprolol tartrate 25-50 mg tab | 20 | 0.70 | 13.97 |
| Midazolam 5 mg/mL; 1 mL | 2 | 1.00 | 2.00 |
| Milrinone 1 mg/mL; 10 mL vial | 0.7 | 55.02 | 38.52 |
| Morphine 10 mg/mL; 1 mL | 0.5 | 0.85 | 0.43 |
| Naloxone 0.4 mg/mL amp | 0.05 | 2.33 | 0.12 |
| Neostigmine 2.5 mg/mL; 1 mL amp | 0.1 | 0.34 | 0.03 |
| Nitroglycerin 0.4 mg/mL; 250 mL bottle | 0.5 | 4.37 | 2.19 |
| Noradrenaline 2 mg/mL; 2mL amp | 2 | 2.02 | 4.04 |
| Ondansetron 4 mg vial | 3 | 0.62 | 1.86 |
| Paracetamol 1,000 mg/100 mL | 4 | 2.87 | 11.49 |
| Paracetamol 500 mg tab | 80 | 0.01 | 0.62 |
| Penicillin 250 mg tab | 20 | 0.03 | 0.54 |
| Phenylephrine 10 mg/mL vial | 2.5 | 8.39 | 20.98 |
| Phytonadione 10 mg/mL amp | 0.05 | 0.23 | 0.01 |
| Potassium Chloride 20% (26.8 mEq); 10mL | 9 | 1.01 | 9.08 |
| Potassium Chloride 8 mEq tab | 10 | 1.46 | 14.55 |
| Promethazine 25 mg vial | 0.05 | 0.16 | 0.01 |
| Propofol 10mg/mL; 20mL vial | 6.5 | 2.79 | 18.16 |
| Protamine 10mg/mL; 5mL vial | 7 | 13.22 | 92.53 |
| Sildenafil 50 mg tab | 0.5 | 0.07 | 0.03 |
| Sodium bicarbonate 7.5%, 20 mL | 2.5 | 1.24 | 3.10 |
| Spironolactone 25 mg tab | 10 | 0.03 | 0.30 |
| Suxamethonium 50 mg/mL; 2 mL amp | 0.1 | 1.86 | 0.19 |
| Tramadol 50 mg tab | 6 | 0.22 | 1.36 |
| Tranexamic acid 500 mg/5 mL amp | 3 | 1.24 | 3.73 |
| Vancomycin 1000 mg vial | 2 | 7.30 | 14.59 |
| Vasopressin 20 units/mL vial | 1 | 21.73 | 21.73 |
| Vecuronium 4 mg/2 mL amp | 2 | 1.94 | 3.88 |
| Verapamil 5 mg/2 mL amp | 0.5 | 6.49 | 3.25 |
| Warfarin 1 mg tab | 5 | 0.05 | 0.27 |
| Warfarin 2 mg tab | 5 | 0.31 | 1.55 |
| Warfarin 5 mg tab | 5 | 0.31 | 1.55 |

*Estimated quantities and costs of medications used for single- and double-valve replacements in Rwanda. Quantity was calculated as an average from six surgical cases. USD= United States dollars.*

Supplementary Table 6: Fluids and costs in US dollars

| Fluid | Quantity | Item cost (USD) | Total Cost (USD) |
| --- | --- | --- | --- |
| D5W; 1000 mL | 5 | 8.81 | 44.50 |
| Lactated ringers; 1000 mL | 10 | 10.52 | 105.20 |
| Normal saline; 50 mL | 22 | 10.39 | 228.58 |
| Normal saline; 100 mL | 7.5 | 9.74 | 73.07 |
| Normal saline; 200 mL | 6 | 10.42 | 62.52 |
| Normal saline; 1000 mL | 5 | 10.72 | 53.60 |

*Estimated quantities and costs of fluids used for single- and double-valve replacements in Rwanda. Quantity was calculated as an average from six surgical cases. USD= United States dollars.*
